# Comparative effectiveness of anti-viral drugs with dual activity for treating hepatitis B and HIV co-infected patients: a network meta-analysis

**DOI:** 10.1186/s12879-018-3506-x

**Published:** 2018-11-14

**Authors:** Cho Naing, Yong Poovorawan, Kew Siang Tong

**Affiliations:** 1 International Medical University, Kuala Lumpur, Malaysia; 2 Division of Tropical Heath and Medicine, James Cook University, Townsville, QLD Australia; 3 Centre of Excellence in Clinical Virology, Faculty of Medicine, Chulalongkorn University, Bangkok, Thailand

**Keywords:** Hepatitis B, HIV, Antiviral, Network meta-analysis

## Abstract

**Background:**

There are randomized trials assessing a variety of antiviral drugs for hepatitis B virus (HBV), but the relative effectiveness of these drugs in the treatment of patients co-infected with human immunodeficiency virus (HIV) remains unclear. The objectives of the current study were to estimate and rank the relative effectiveness of antiviral drugs for treating HBV and HIV co-infected patients.

**Methods:**

Randomized trials, assessing the efficacy of antiviral drugs for HBV and HIV co-infected patients were searched in health-related databases. The methodological quality of the included trials was evaluated using the Cochrane risk of bias tool. Main outcome in this meta-analysis study was the success of treatment by antivirals as determined by virologic response. We performed pairwise and network meta-analysis of these trials and assessed the quality of evidence using the GRADE approach.

**Results:**

Seven randomized trials (329 participants) were included in this network meta-analysis study. A network geometry was formed with six treatment options including four antiviral drugs, adefovir (ADV), emtricitabine (FTC), lamivudine (LMV) and tenofovir disoproxil fumarate (TDF), combination treatment of TDF plus LMV, and placebo. The weighted percentage contributions of each comparison distributed fairly equally in the entire network of evidence. An assumption of consistency required for network meta-analysis was not violated (the global Wald test for inconsistency: Chi^2^(4) = 3.63, *p* = 0.46). The results of estimates showed no differences between the treatment regimens in terms of viral response for treating HBV and HIV co-infected patients, which spanned both benefit and harm (e.g. LMV vs TDF plus LMV: OR: 0.37, 95%CI: 0.06–2.41). Overall, the certainty of evidence was very low in all comparisons (e.g. LMV vs TDF plus LMV: 218 fewer per 1000,121 more to 602 fewer, very low certainty). Therefore, we remained uncertain to the true ranking of the antiviral treatments in HBV/ HIV co-infected patients.

**Conclusions:**

The findings suggest that the evidence is insufficient to provide guidance to the relative effectiveness of currently available antiviral drugs with dual activity in treating co-infection of HBV/HIV. Well-designed, large clinical trials in this field to address other important outcomes from different epidemiological settings are recommended.

**Electronic supplementary material:**

The online version of this article (10.1186/s12879-018-3506-x) contains supplementary material, which is available to authorized users.

## Background

Human immunodeficiency virus (HIV) is an RNA virus and hepatitis B virus (HBV) is a partially double stranded DNA virus, both of which share common modes of transmission. Studies have reported that co-infection with HBV and HIV-1 is common [1–3]. Among the 40 million persons infected with HIV worldwide, an estimated 2–4 million are co-infected with HBV [4], albeit with variation in age-specific prevalence, geographic distribution and the predominant routes of transmission [4, 5]. Hence, the treatment of chronic HBV in HIV-infected individuals with dual antiretroviral therapy (ART) requires careful consideration.

In co-infected patients, HIV accelerates the progression of HBV-related liver disease. It has been reported that HIV infection has negative impact on the natural history of HBV infection leading to increased rates of persistent infection, higher HBV DNA levels, lower rates of hepatitis Be antigen loss, increased cirrhosis and liver-related mortality, and increased risk of hepatocellular carcinoma (HCC) at lower CD4+ T cell counts [6]. The aim of treating HBV infection is to prevent patients from progressing to the chronic stage [7, 8].

Thus far, emtricitabine (FTC), lamivudine (LMV), tenofovir disoproxil fumarate (TDF) and tenofovir alafenamide (TAF) have demonstrated dual activity for HBV and HIV [9]. However, these drugs vary in therapeutic activity and toxicity. There are randomized trials (RCTs) assessing a variety of antiviral drugs for HBV, but their relative effectiveness in the treatment of patients co-infected with HBV and HIV remains unclear. A network meta-analysis permits inferences into the comparative effectiveness of interventions from direct comparisons (i.e., treatments are directly compared within an RCT) and indirect comparisons (i.e., treatments are compared between RCTs by combining results against a common comparator treatment) and it also allows ranking of these different treatments [10, 11]. Overall, the objectives of the current study were to estimate and rank the relative effectiveness of antiviral drugs for treating HBV and HIV co-infected patients.

## Methods

The current study was performed in accordance with the preferred reporting items for network meta-analyses (PRISMA-NMA) [12]. A protocol of this study is available in PROSPERO (CRD42016035539).

### Search

Relevant RCTs were searched in the health-related databases of Ovid MEDLINE, Ovid Embase, The Cochrane Library, and Google Scholar. The search strategies for the Ovid MEDLINE are given in Additional file 1. Our search was restricted to publications in the English language up to February 2018. To identify ongoing or completed trials, we also searched in ClinicalTrials.gov (http://www.clinicaltrials.gov/), WHO International Clinical Trials Registry Platform (http://apps.who.int/trialsearch/Default.aspx) and EU Clinical Trials Register (https://www.clinicaltrialsregister.eu/). We did not search for the adverse effects of antiviral drug interventions separately and only used data contained in the publications identified for the present study.

### Study selection

Individual studies were selected based on the PICOS format [13]:

#### Study Population (P)

Patients co-infected with HBV and HIV, regardless of gender, age, the severity of infections and HBV genotype were included.

#### Interventions (I)

Antiviral drugs (monotherapy or combination treatment) for the treatment of HBV infection were considered.

#### Comparisons (C)

Data from an antiviral drug versus an alternative antiviral drug, combination treatment, or placebo were included.

#### Study Outcomes (O)

Treatment success (virologic response) was defined as achieving undetectable levels of HBV DNA in patients at the end of 1 year (36–52 weeks)**.** This represents the suppression of HBV DNA levels as stated in the primary studies on the scheduled follow-up, regardless of their HBeAg status. Adverse events /serious adverse events were as stated in the included studies. For example, hepatic failure after the commencement of therapy was defined as an increase in ALT to > 3-5x the upper limit of normal from a baseline value below this level [14, 15].

#### Study design (S)

RCT.

Studies which did not meet the inclusion criteria were excluded.

### Data extraction

Two review authors independently screened the titles and abstracts of citations from the electronic database search and retrieved full-text of all potentially relevant articles. When studies had duplicate publications, we extracted the maximum amount of data from the available publications. The two review authors then independently checked the full-text articles for eligibility based on the inclusion criteria. The two reviewers extracted data from the included studies using a piloted data extraction sheet. Data collected were authors, country, publication year, participant’s characteristics, details of intervention and controls regimen (dosage, route of administration, frequency and duration), outcomes, method of outcome measurements, follow-up time points of the outcome and adverse events. Any discrepancy between the two investigators was resolved by discussion and consensus.

### Methodological quality assessment

The methodological quality of the included trials was evaluated, using the Cochrane risk of bias tool [16]. We checked three domains for the risk of bias assessment such as adequate sequence generation, allocation concealment, blinding of participants and outcome assessors for each trial for the virologic response. As there was only one outcome in the current review, the risk of bias assessments at the outcome level was applied to the whole study. We used these ratings to further Grading of Recommendations Assessment, Development, and Evaluation (GRADE) assessment for risk of bias category accordingly [17].

### Data synthesis

Main outcome in this review was success of treatment by HBV antiviral treatment as determined by virologic response. The intention-to-treat (ITT) analysis was used for efficacy assessments.

#### Pairwise comparison

If the studies included had been reported in similar ways (i.e. the comparators, the outcomes reported and follow-up time point), then direct pairwise meta-analyses of head-to-head comparisons were performed using standard frequentist approaches. We used odds ratio (OR) along with 95%confidence interval (CI) for the dichotomous variable as a measure of the strength of association between the treatment used and efficacy/adverse events. We pooled ORs with a DerSimonian-Laird random-effects model, as the heterogeneity was high (*I*^2^ > 50%) [13]. Initially, we planned to perform a sensitivity analysis to investigate the potential impact of studies at high risk of bias in the pooled studies. We also planned to do stratified analysis by prior LMV naive or LMV experienced. However, the number of studies identified was too small to carry out these analyses. Publication bias was not assessed with the contoured-enhanced funnel plot because the number of included studies was fewer than the recommended 10 included studies [13]. We assumed the risk of publication bias may be higher as the estimates were based on small RCTs [18].

#### Network meta-analyses

We performed network meta-analyses within a frequentist framework using random-effects models [10, 19, 20]. The approach incorporated both direct and indirect information through the use of a common comparator to obtain estimates of the relative interventional effects on multiple intervention comparisons. We established network connections. The percentage contribution of each estimate to the entire network was calculated. The results were presented as contribution plots in which the weighted squares indicated the percentage contribution of each comparison [21].

We investigated network inconsistency with the use of the global Wald test for inconsistency [22]. The network meta-analysis results were reported for ‘mixed treatment contrasts’, including both direct and indirect evidence from across the entire network [21, 22]. For a ranking of the effectiveness and the uncertainty, probability values were summarised and reported as ‘Surface Under the Cumulative Ranking Curve’ (SUCRA), as described elsewhere [11, 19]. SUCRA = 1 or 0, if an antiviral drug intervention ranked first or last, respectively. Statistical significance was set at *p* value ≤0.05.

### Assessing the quality of evidence

We assessed the quality of evidence derived from the pairwise and network meta- analysis, following the GRADE approach described elsewhere [17, 20, 23, 24]. We rated direct evidence from RCTs, using the standard GRADE approach on the five categories such as study limitations (risk of bias), precision, consistency of results, directness of evidence and publication bias. On these five categories, we judged the overall confidence in estimates of effect for virologic responses for each direct comparison as ‘high’, ‘moderate’, ‘low’ or ‘very low’ quality of evidence. For indirect comparison, we rated evidence from the most dominant first-order loop by first taking the lowest certainty of direct comparisons. We considered further rating down, if there were concerns with intransivity [24]. For NMA mixed estimates, we started with the higher quality of the two certainty ratings and rated down certainty for incoherence (degree of inconsistency between direct and indirect effect estimates) in the final quality rating [23, 24]. We did not rate down intransivirty [11] in the current study as there was no important imbalance in the distribution of effect modifiers (e.g. age, gender, dosage) across seven studies. Pairwise meta-analysis was done with RevMan 5.3 (*The Nordic Cochrane Centre, The Cochrane Collaboration*), while network meta-analysis was with STATA 15.0 (StataCorp, TX).

## Results

### Trials included

**Fig. 1:**
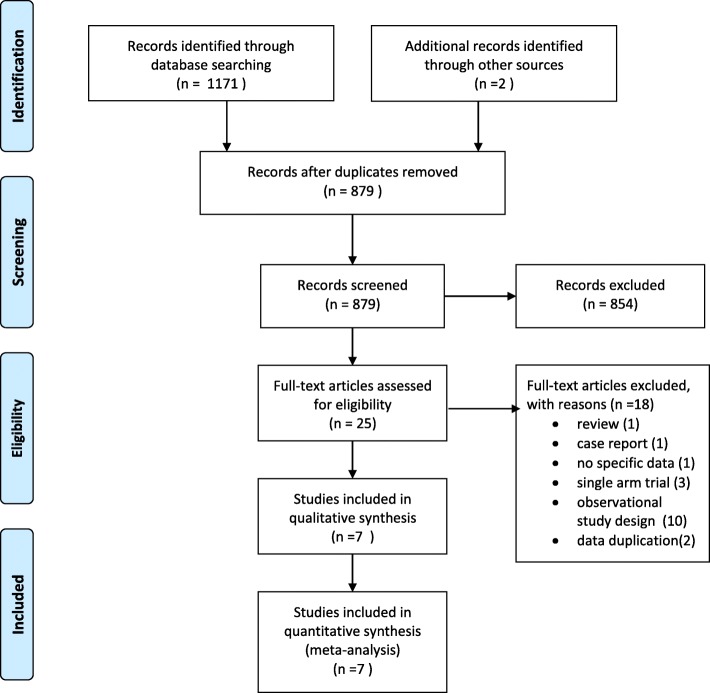
Study selection PRISMA flowchart

Figure 1 shows a four-phase study selection process. The initial search yielded 1171 citations. After the title and abstract screening, 25 full-text papers were reviewed and a final of 7 studies (*n* = 329) were included in this review [14, 15, 25–29]. The largest comparison was LMV versus placebo (*n* = 122, 32.4%), followed by LMV versus TDF plus LMV (*n* = 116, 31%). A summary of the 18 excluded studies [2, 6, 7, 30–44] is provided in Additional file 2: Table 1 presents the characteristics of the included studies. The number of participants ranged from 12 [25] to 122 [14]. Three RCTs were multi-country studies [14, 25, 27], two RCTs were from China [28, 29] and one RCT each was from Thailand [15] and the USA [26]. Six studies were two-arm RCTs [14, 15, 25, 26, 28, 29], and only one study was a three-arm RCT [27]. The distribution of studies and comparisons are presented in Additional file 3. The risk of bias was affected by inadequate information on the allocation concealment and the blinding status of the RCTs included (Additional file 4).

**Table 1:** Characteristics of the studies included in the network meta-analysis

Study [Reference No]	Country	RCT design	Total samples	Age in year (SD or range)	Males	Comparators	Time point in week	Diagnosis of HBV	Main mode of transmission	Remarks
Dore, 1999 [14]	Canada, Australia, Europe, & South Africa (CAESAR)	2 arms; ITT	122	37^a^(range: 22–70)	96%	LMV (150 mg twice/D) vs placebo	4, 8, 12, 20, 28, 36, 44, 52.	Amplicor PCR (Roche, NJ)		68% ART received
Dore, 2004 [25]	Western EU, North America, Australia	2 arms; as treated	Gr 1: 12 Gr2:11	Gr 1: 40^a^ Gr2:42^a^	100%^a^	Gr1: TDF (300 mg/D) vs placebo. Gr 2:TDF (300 mg/D) vs TDF + LMV (150 mg twice/D).	12,24,48	HBV DNA (Roche Amplicor),		A sub-study of 908 ART exp. (Gr1) & 903 naive (Gr2); ART naïve group & ART experienced group
Peters, 2006 [26]	USA	2 arms; ITT	52	47^a^	24%	ADV vs TDF	12,24,36,48	Roche Amplicor CobasPCR	IVDU:13.5%	stop early after interim results
Mathews, 2008 [27]	3 countries; Netherlands, Australia & Thailand (NAT)	3 arms; ITT	36	35.5 (SD:±8.4)	64%	LMV (150 mg twice/D); TDF(300 mg/D)	12,24,48	Versant HBV DNA 3.0 bDNA assay (Bayer HealthCare, NY); COBAS TaqMan HBV Test (Roche Diagnostics NJ).	Hetero (78%)	ART naiïve; EFV (600 mg/D) to all 3 groups; 7 (19%) with AIDS
Avihingsanon,2010 [15]	Thailand	2 arms; ITT	16	34^a^(range: 30–39)	12%	TDF + FTC (600 mg + 300 mg/D) vs FTC (300 mg/D)	12,24,48	Versant HBV DNA 3.0 bDNA assay (Bayer HealthCare, NY); COBAS TaqMan HBV Test (Roche Diagnostics NJ).	Hetero (75%)	EFV (600 mg single dose/D)
Gu, 2014 [28]	China	2 arms; ITT	50	36 (SD:±9.5)	88%	TDF+ LMV vs LMP	12,48,96	COBAS Ampliprep/COBAS TaqMan	86% hetro + homo: 51.2% MSM	ART received
Wang, 2016 [29]	China	2 arms;ITT	80	29^a^(range: 24–36)	0%	TDF + LMV vs LMV	36	m2000 RT System (Abbott RT HBV Assay, California),	_	pregnant women

*ART* antiretroviral therapy, *D* day, *exp*. experienced, *hetro* hetrosexuial contacts, *homo* homosexual contacts, *ITT* intention-to-treat analysis, *IVDU* intravenous drug users, *MSM* men who have sex with men, *RT* RealTime, *SD* standard deviation, *SVR* suppression of viral loads, *yr.* year; ^a^medium; ^$$^median value (range or IRQ values are not provided)

### Six-node analysis

**Fig. 2:**
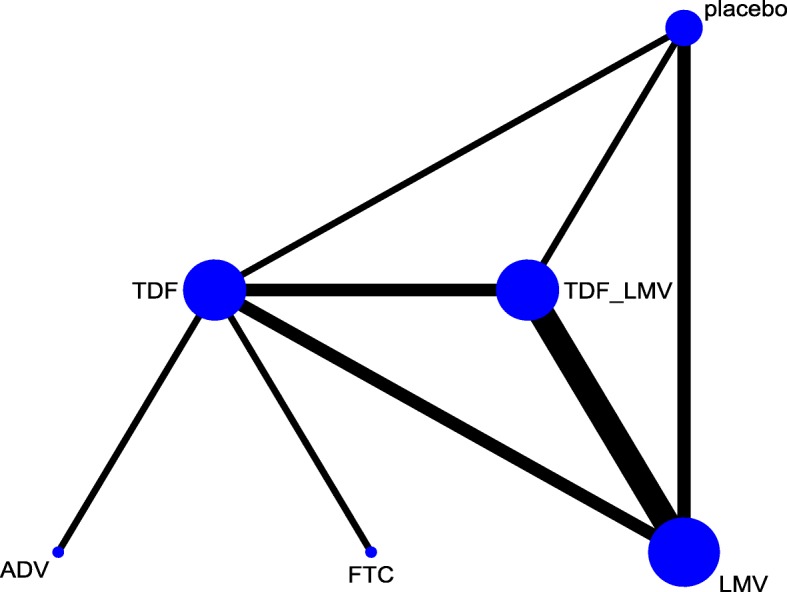
Network map of different antiviral drugs for treating hepatitis B and HIV co-infected patients

**Fig. 3:**
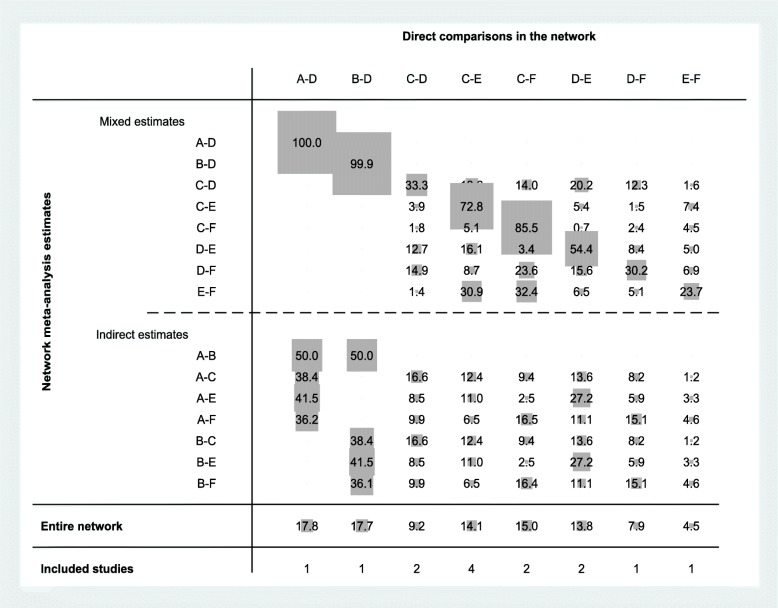
Contributing plot

Figure 2 shows a network geometry of treatment success and provides eight direct (*n* = 329) and seven indirect comparisons for six treatment options. Six treatment options included were five antiviral drugs such as adefovir dipivoxil (ADV), FTC, LMV, TDF, TDF plus LMV and placebo. The contributing plot indicates the contribution of each direct comparison to indirect and network estimates (Fig 3). The weighted percentage contributions of each comparison were distributed fairly equally in the entire network of evidence. The global Wald test suggested the presence of consistency in the network [Chi^2^ (4) = 3.63, *p* = 0.46].

**Fig. 4:**
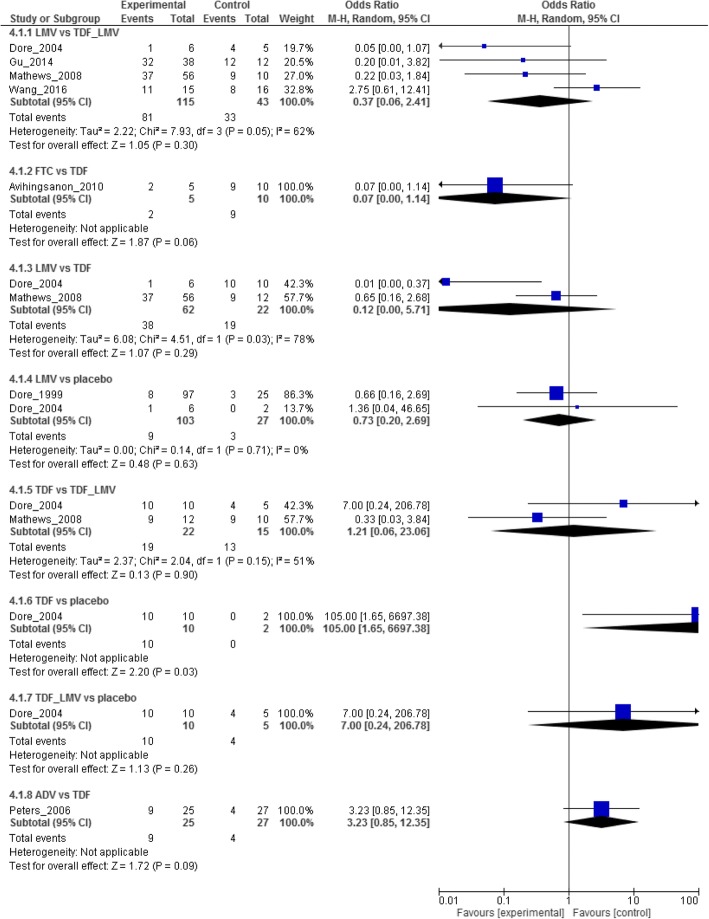
Forest plot of pairwise meta-analysis

**Fig. 5:**
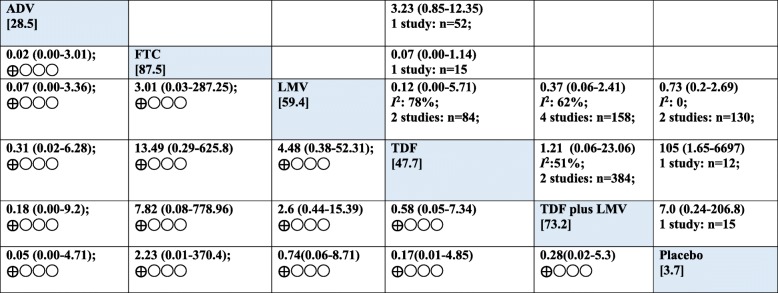
Results of pairwise meta-analyses and network meta-analysis with consistency model for antiviral treatment

Pairwise analysis of the relative efficacy of antiviral for viral suppression in HBV/HIV was reported from eight pairwise comparisons. The result is very low certainty of evidence that there were no differences between the treatment regimens in virologic response and wide 95%CIs, which spanned both benefit and harm (Fig 4). For instance, four studies compared LMV with TDF plus LMV. There was no difference between the two regimens in virologic response (LMV vs TDF plus LMV (OR 0.37, 95%CI: 0.06–2.41; 218 fewer (121 more to 602 fewer) per 1000 patients, very low certainty). A single trial reported no difference between FTC and TDF in virologic response for the co-infected patients (FTC vs TDF (OR 0.07, 95%CI: 0.00–1.14; 166 more (199 more to 310 fewer) per 1000 patients, very low certainty] (Table 2). The direct, indirect and network (mixed) comparisons for the six-node comparison after completing assessment for GRADE criteria are presented in Tables 2, 3 and Fig 5.

**Table 2:** GRADE quality assessment of direct evidence of each pairwise treatment comparison for treatment success

Treatment comparison	Number of head-to head trials(n)	Study limitations`	Precision	Consistency	Directness	Publication bias	Direct Estimate; OR (95% CI)	Absolute effect per 100 treated (95% CI)	Overall quality of evidence
LMV vs TDF plus LMV	4 (158)	serious^a^	very serious^b^	very serious (*I*^*2*^: 62%)^c^	serious	likely to exist	0.37 (0.06–2.41)	218 fewer (from 121 more to 602 fewer)	very low ⊕◯◯◯
FTC vs TDF	1 (15)	serious^a^	very serious^a^	not serious	not serious	likely to exist	0.07 (0.00–1.14)	513 fewer (0 to 11 more)	very low ⊕◯◯◯
LMV vs TDF	2 (84)	serious^a^	very serious^a^	very serious^c^(*I*^*2*^: 78%)	serious	likely to exist	0.12 (0.00–5.71)	27 fewer (87 fewer to 141 more)	very low ⊕◯◯◯
LMV vs placebo	2 (130)	serious^a^	very serious^a^	not serious (*I*^*2*^: 0%)	serious	likely to exist	0.73 (0.2–2.69)	432 fewer (0 to 109 more)	very low ⊕◯◯◯
TDF vs TDF plus LMV	2 (37)	serious^a^	very serious^a^	very serious^c^(*I*^*2*^: 51%)	serious	likely to exist	1.21 (0.06–23.06)	21 more (127 more to 586 fewer)	very low ⊕◯◯◯
TDF vs placebo	1 (12)	serious^a^	very serious^b^	not serious	not serious	likely to exist	105.00(1.65–6697.38)	0 fewer (0 fewer to 0 fewer)	very low ⊕◯◯◯
TDF plus LMV vs placebo	1 (15)	serious^a^	very serious^b^	not serious	not serious	likely to exist	7.00 (0.24–206.78)	239 more (294 more to 341 fewer)	very low ⊕◯◯◯
ADV vs TDF	1 (52)	serious^a^	very serious^b^	not serious	not serious	likely to exist	3.23 (0.85–12.35)	212 more (19 fewer to 534 more)	very low ⊕◯◯◯

For domains “Study Limitations”, “Precision”, “Consistency”, and “Directness”: rated as not serious, serious, or very serious issues. For the domain “Publication bias”: not likely or likely to exist. Reasons are provided when rating down. All direct comparisons begin with a “High” rating. ^a^Rated down one level for risk of bias as study limitations;^b^Rated down two levels for imprecision; ^c^Rated down two levels for substantial heterogeneity based on *I*^*2*^ values; *CI* Confidence interval, *OR* Odds ratio. *ADV* Adefovir, *FTC* Emtricitabine, *LMV* Lamivudine, *TDF* Tenofovir disoproxil fumarate, *TDF plus LMV* Combined Tenofovir disoproxil fumarate and Lamivudine

**Table 3:** Network estimates of treatment success with 95% confidence intervals and GRADE assessments

Treatment comparison	Direct estimate; OR (95% CI)	quality of evidence	Indirect estimate; OR (95% CI)	quality of evidence	Network estimate; OR (95% CI)	quality of evidence
ADV FTC	Not available	Not available	0.02 (0.00–3.01)	Very low ⊕◯◯◯	0.02 (0.00–3.01)	Very low ⊕◯◯◯
ADV vs LMV	Not available	Not available	0.07 (0.00–3.36)	Very low ⊕◯◯◯	0.07 (0.00–3.36)	Very low ⊕◯◯◯
ADV vs TD	Not available	Not available	0.31 (0.02–6.28)	Very low ⊕◯◯◯	0.31 (0.02–6.28)	Very low ⊕◯◯◯S
ADV vs TDF plus LMV	Not available	Not available	0.18 (0.00–9.2)	Very low ⊕◯◯◯	0.18 (0.00–9.2)	Very low ⊕◯◯◯
ADV vs placebo	Not available	Not available	0.05 (0.00–4.71)	Very low⊕◯◯◯	0.05 (0.00–4.71)	Very low ⊕◯◯◯
FTC vs LMV	Not available	Not available	3.01 (0.03–287.25)	Very low ⊕◯◯◯	3.01 (0.03–287.25)	Very low ⊕◯◯◯
FTC vs TDF plus LMV	Not available	Not available	7.82 (0.08–778.9)	Very low	7.82 (0.08–778.9)	Very low
FTC vs placebo	Not available	Not available	2.23 (0.01–370.4)	Very low ⊕◯◯◯	2.23 (0.01–370.4)	Very low ⊕◯◯◯

*ADV* Adefovir, *FTC* Emtricitabine, *LMV* Lamivudine, *TDF* Tenofovir disoproxil fumarate, *TDF plus LMV* Combined Tenofovir disoproxil fumarate and Lamivudine. Rated down one level for incoherence

Treatment relative ranking in the network meta-analysis is presented in Additional file 5. FTC had the highest probability of being the best choice for treating patients co-infected with HBV/HIV, with the TDF plus LMV combination being the second best. The SUCRA and ranking results are under the circumstances of small number of the included studies and wide estimates ranging from benefit to harm. The evidence on which the SUCRA rankings are warranted was of very low quality and therefore untrustworthy [45]. We made overall evidence in view of the GRADE approach rather than the SUCRA rankings (Fig 5).

Overall, there was very low certainty evidence whether any antiviral regimens included in this study were better in virologic response for treatment of HBV/HIV co-infected patients since the certainty of the evidence was assessed as very low.

### Other outcomes

Due to the small number of studies, other outcomes limited the summary estimates. Two studies had reported that HBV DNA was significantly decreased after treatment [14, 28]. With regards to the adverse events/serious adverse events, three studies included in this review had reported hepatic flare or ALT flare after initiation of antiviral treatments. Due to variations in antiviral drugs administered and/or inconsistences in reporting, it was difficult to make the pooled analysis. The prevalence of hepatic flare after initiation of antiviral treatments was 54.5% (6/11) [25], while this was 21.2% (11/52) in one trial [26] and 19% (3/16) in another trial [15]. These events were resolved, and none was associated with the development of hepatic decomposition [15, 25, 26].

## Discussion

### Summary of main results

In this network meta-analysis, we have combined direct and indirect evidence on the relative efficacy of antiviral drugs interventions at the end of maximum 1 year treatment. The results of this NMA provide very low quality evidence that no regimen provided better rates of treatment success.

The use of virologic responses to represent suppression of HBV DNA after giving antiviral treatments served as surrogate outcomes in this review was clinically relevant. The main goal of the treatment of HBV infection was the sustained suppression of HBV replication because suppression is associated with a normalization of transaminase levels and improvement in histologic findings [6, 8].

There was only one study evaluating the efficacy of FTC and showed no difference in virologic response than the comparators. Also, any regimen in the current study was no different from the comparators and there were wide CIs. In the GRADE approach, it was rated down two levels for imprecision. Overall, the evidence was very low quality. Hence, the present findings were limited in definitively resolving the question of optimal treatment choice. Studies have reported that treatment responses to TDF plus LMV combination therapy would be likely to be mediated by TDF alone as LMV may have no or minimal antiviral efficacy in the presence of LMV-resistant HBV mutants [46]. A study had reported that TDF monotherapy could maintain effective viral suppression over up to 288 weeks of continuous therapy without the selection of TDF resistance [47]. The fact that the LMV monotherapy was ranked higher than the TDF monotherapy as found in this review was an unexpected outcome and this was very low certainty of evidence. Studies have reported that 3TC (LMV) resistant HBV emerged in about 40 and 90% of patients after 2 and 4 years on 3TC respectively [48], while TDF had a high resistance barrier with no resistance identified to date after up to 6 years of monotherapy for chronic hepatitis B [49]. A systematic review had highlighted that entecavir and TDF were most effective in HBeAg-positive treatment-naive patients, while TDF was most effective in HBeAg negative treatment-naive patients in all surrogates outcomes at the end of first year of therapy [50].

In published reviews, TDF demonstrated the most effective viral treatment for patients with chronic HBV [50, 51]. This was not observed in this study on patients with dual HBV/HIV infection. This implied that TDF is less potent for suppression of HIV DNA. A concern is that suppression of HBV DNA to undetectable levels was determined in the study population with continuing antiviral therapy. Hence, the stability of treatment response, after stopping therapy has not yet been investigated.

### Study limitations

Due to a small number of studies included in this review for direct comparison, there was inadequate statistical power to detect significant differences among the drugs evaluated. Moreover, the currently recommended limit of serum HBV-DNA diagnostic assays is 20 IU/ml or 10 IU/ml (around 100 copies/ml or 50 copies/ml) on the scheduled follow-up regardless of their HBeAg status. The studies included in this analysis used different threshold for the definition of virological suppression. For instance, it was a serum HBV-DNA < 1000 copies/ml in one study [25], while other studies used < 400 copies/ml [14] or < 200 copies/ml [26]. Due to the use of higher cut-off for quantification of serum HBV-DNA diagnostic assays in the primary studies, the misclassification bias is a concern. An interpretation of the findings, therefore, needs a caution regarding such bias.

Studies in non-English language may have been missed. Telbivudine is licensed for HBV and HIV treatment, but none of the included studies assessed this antiviral drug. As our focus was on clinical effectiveness only, the cost-effectiveness of preferentially using a particular drug intervention was not done and should be examined in future studies. The highest prevalence was observed in sub-Saharan Africa and East Asia, where 5–10% of the adult population was chronically infected with HBV [52, 53]. But, none of the RCTs were from sub-Saharan Africa, indicating a geographical imbalance and an interpretation of this review was limited with regard to generalizability. Available data was sufficient to summarize only one surrogate clinical outcome. Currently, there is no agreement on the most appropriate surrogate markers of a long-term outcome or even of the validity of on-treatment measurements [49].

Nevertheless, the outcome used in this review was reasonable; studies have shown that for treatment with LMV, telbivudine, and ADV, subsequent resistance is low for those whose viral load is maintained at less than 1000 copies/m [54].

### Clinical implications

The escalating HIV burden in some countries with a background prevalence of chronic HBV infection will contribute to an increasing number of people with HIV-1/HBV co-infection in the affected countries. Thus, an antiviral agent with dual efficacy against HIV-1/HBV in combination with at least one other antiretroviral agent could provide a cost-effective approach to manage these two chronic viral infections in resource-limited countries [14].

## Conclusion

The findings suggest that there was insufficient evidence to provide the relative effectiveness of currently available antiviral drugs with dual activity in treating co-infection of HBV/HIV. Future well-designed, large clinical trials in this field to address other important outcomes from different geographical settings are recommended.

## Additional files

Additional file 1: Citations and Ovid MEDLINE. (DOC 44 kb)

Additional file 2: Excluded studies and reasons for exclusion. (DOC 38 kb)

Additional file 3: Distribution of studies and comparisons. (DOC 40 kb)

Additional file 4: Risk of bias assessment by the review authors. (DOC 33 kb)

Additional file 5: Treatment relative ranking. (DOC 31 kb)

## Abbreviations

ADV: Adefovir
ART: Antiretroviral therapy
CENTRAL: Cochrane Central Register of Controlled Trials
CI: Confidence interval
FTC: Emtricitabine
GRADE: Grading of Recommendations Assessment, Development, and Evaluation
HBsAg: Hepatitis B surface antigen
HBV: Hepatitis B virus
HCC: Hepatocellular carcinoma
HIV: Human immunodeficiency virus
LMV: Lamivudine
NMA: Network meta-analysis
OR: Odds ratio
PRISMA-NMA: PRISMA Extension Statement for Reporting of Systematic Reviews Incorporating Network Meta-analyses of Health Care Interventions
RCT: Randomized trials
SUCRA: Surface under the cumulative ranking curve
TDF: Tenofovir disoproxil fumarate
