# Supplementary material for: Comparative effectiveness of anti-viral drugs with dual activity for treating hepatitis B and HIV co-infected patients: a network meta-analysis
Source: BMC Infect Dis. 2018 Nov 14;18:564. doi: 10.1186/s12879-018-3506-x (PMC6234602; doi:10.1186/s12879-018-3506-x)
Supplement: Supplementary file 1 — Citations and Ovid MEDLINE. (DOC 44 kb) [file 12879_2018_3506_MOESM1_ESM.doc]

**Additional File 1**. Citations and Ovid MEDLINE(R) <1946 to Present>

| Search terms**:** 1 | "randomized controlled trial".pt. (513377) |
| --- | --- |
| 2 | (random$ or placebo$ or single blind$ or double blind$ or triple blind$).ti,ab. |
| 3 | (retraction of publication or retracted publication).pt. |
| 4 | 1 or 2 or 3 |
| 5 | (animals not humans).sh. |
| 6 | ((comment or editorial or meta-analysis or practice-guideline or review or letter or journal correspondence) not "randomized controlled trial").pt. |
| 7 | (random sampl$ or random digit$ or random effect$ or random survey or random regression).ti,ab. not "randomized controlled trial".pt. |
| 8 | 5 or 6 or 7 |
| 9 | 4 not 8 |
| 10 | (random$ or placebo$ or single blind$ or double blind$ or triple blind$).ti,ab. |
| 11 | RETRACTED ARTICLE/ |
| 12 | 10 or 11 |
| 13 | (animal$ not human$).sh,hw. |
| 14 | (book or conference paper or editorial or letter or review).pt. not exp randomized controlled trial/ |
| 15 | (random sampl$ or random digit$ or random effect$ or random survey or random regression).ti,ab. not exp randomized controlled trial/ |
| 16 | 13 or 14 or 15 |
| 17 | 12 not 16 |
| 18 | 9 or 17 |
| 19 | exp hepatitis B/HIV |
| 20 | ((HBV$)).tw. |
| 21 | exp hepatitis infection/ |
| 22 | 19 or 20 or 21 |
| 23 | exp Drug Therapy/ |
| 24 | (antiviral and therap*).mp. [mp=ti, ab, sh, hw, tn, ot, dm, mf, dv, kw, nm, kf, ps, rs, ui] |
| 25 | (TDF* or LMP*or ADV* or FTC* |
| 26 | 23 or 24 or 25 |
| 27 | 18 and 22 and 26 |
| 28 | limit 27 to english language |
| 29 | limit 28 to human |
| 30 | remove duplicates from 29 |
