# Supplementary material for: Comparative effectiveness of anti-viral drugs with dual activity for treating hepatitis B and HIV co-infected patients: a network meta-analysis
Source: BMC Infect Dis. 2018 Nov 14;18:564. doi: 10.1186/s12879-018-3506-x (PMC6234602; doi:10.1186/s12879-018-3506-x)
Supplement: Supplementary file 2 — Excluded studies and reasons for exclusion. (DOC 38 kb) [file 12879_2018_3506_MOESM2_ESM.doc]

Additional File 2. Excluded studies and reasons for exclusion

| Author [Ref. no] | Reason |
| --- | --- |
| Thio, 2006 [2] | review |
| Thio , 2009 [6] | review |
| Aggarwal,2004 [7] | not a drug efficacy study |
| Hoffmann, 2008[30] | observational study design |
| Gutierrez,2008 [31] | case report |
| Lacombe,2008 [32] | observational study design |
| Nuesch, 2008 [33] | observational study design |
| Alvarez-Uria,2009[34] | observational study design |
| Idoko,2009 [35] | observational study design |
| Engell,2011 [36] | observational study design |
| Matthews, 2011 [37] | subset of an included trial |
| Nelson, 2012 [38] | no separate data for HBV/HIV |
| Matthews, 2013 [39] | subset of an included trial |
| Kang,2014 [40] | observational study design |
| Miailhes, 2014 [41] | single arm study with no comparator |
| Yu, 2014 [42] | observational study design |
| Lee et al. 2015 [43] | observational study design |
| Li, 2016 [44] | observational study design |
