# Supplementary material for: Comparative effectiveness of anti-viral drugs with dual activity for treating hepatitis B and HIV co-infected patients: a network meta-analysis
Source: BMC Infect Dis. 2018 Nov 14;18:564. doi: 10.1186/s12879-018-3506-x (PMC6234602; doi:10.1186/s12879-018-3506-x)
Supplement: Supplementary file 3 — Distribution of studies and comparisons. (DOC 40 kb) [file 12879_2018_3506_MOESM3_ESM.doc]

Additional File 3. Distribution of studies and comparisons

| Study [Ref. no] | Treatment | Events | Total |
| --- | --- | --- | --- |
| Dore,1999 [14] | LMV | 8 | 97 |
| Dore,1999 [14] | Placebo | 3 | 25 |
| Dore,2004 [25] | LMV | 1 | 6 |
| Dore,2004 [25] | TDF plus LMV | 4 | 5 |
| Dore,2004 [25] | TDF | 10 | 10 |
| Dore,2004 [25] | Placebo | 0 | 2 |
| Peters,2006 [26] | TDF | 4 | 27 |
| Peters,2006 [26] | ADV | 9 | 25 |
| Mathews,2008 [27] | TDF | 9 | 12 |
| Mathews,2008 [27] | LMV | 37 | 56 |
| Mathews,2008 [27] | TDF plus LMV | 9 | 10 |
| Avihingsanon,2010 [15] | TDF | 9 | 10 |
| Avihingsanon,2010 [15] | FTC | 2 | 5 |
| Gu,2014 [28] | LMV | 32 | 38 |
| Gu,2014 [28] | TDF plus LMV | 12 | 12 |
| Wang,2016 [29] | LMV | 11 | 15 |
| Wang,2016 [29] | TDF plus LMV | 8 | 16 |

ADV:Adefovir; FTC: emtricitabine; LMP: Lamivudine; TDF: Tenofovir disoproxil fumarate.
