# Supplementary material for: Comparative effectiveness of anti-viral drugs with dual activity for treating hepatitis B and HIV co-infected patients: a network meta-analysis
Source: BMC Infect Dis. 2018 Nov 14;18:564. doi: 10.1186/s12879-018-3506-x (PMC6234602; doi:10.1186/s12879-018-3506-x)
Supplement: Supplementary file 4 — Risk of bias assessment by the review authors. (DOC 33 kb) [file 12879_2018_3506_MOESM4_ESM.doc]

Additional File 4. Risk of bias assessment by the review authors

| Study [Ref. No.] | Randomization | Allocation concealment | Blinding |
| --- | --- | --- | --- |
| Dore, 1999 [14] | Low | Unsure^¶^ | Low^*^ |
| Dore, 2004 [25] | Low | Unsure^¶^ | High^**^ |
| Peters, 2006 [26] | Low | Low | Low |
| Mathews, 2008 [27] | Low | Low | Unsure |
| Avihingsanon, 2010 [15] | Low | Unsure | Unsure |
| Gu, 2014 [28] | Low | Unsure | Unsure |
| Wang, 2016 [29] | Low | Unsure | Unsure |

Low: Low risk of bias; High: Low risk of bias; Unsure: Not sure to decide whether low or high risk of bias;

^1*^double blind trial; ^**^ open-label after 24 weeks.

¶randomly allocated, but further description of allocation was not provided in the CAESAR protocol.
