# Supplementary material for: Comparative effectiveness of anti-viral drugs with dual activity for treating hepatitis B and HIV co-infected patients: a network meta-analysis
Source: BMC Infect Dis. 2018 Nov 14;18:564. doi: 10.1186/s12879-018-3506-x (PMC6234602; doi:10.1186/s12879-018-3506-x)
Supplement: Supplementary file 5 — Treatment relative ranking. (DOC 31 kb) [file 12879_2018_3506_MOESM5_ESM.doc]

Additional File 5. Treatment relative ranking

| Treatment | SUCRA^§^ | Probability of being the best | Mean rank |
| --- | --- | --- | --- |
| ADV | 28.5 | 1.1 | 4.6 |
| FTC | 87.5 | 65.8 | 1.6 |
| LMV | 59.4 | 9.2 | 3.0 |
| TDF | 47.7 | 0.9 | 3.6 |
| TDF plus LMP | 73.2 | 22.8 | 2.3 |
| Placebo | 3.7 | 0.3 | 5.8 |

^§^ Surface Under the Cumulative Ranking Curve; Abbreviations: ADV: Adefovir; FTC: Emtricitabine; LMV: Lamivudine; TDF: Tenofovir disoproxil fumarate.

SUCRA values range from 0 to 100%. The higher the SUCRA value, the closer to 100%, the higher the likelihood that a therapy is in the top rank or one of the top ranks; the closer to 0 the SUCRA value, the more likely that a therapy is in the bottom rank, or one of the bottom ranks.
